# Spectral characterization, antioxidant, antimicrobial, cytotoxic, and cyclooxygenase inhibitory activities of *Aloysia citriodora* essential oils collected from two Palestinian regions

**DOI:** 10.1186/s12906-021-03314-1

**Published:** 2021-05-17

**Authors:** Nidal Jaradat, Mohammed Hawash, Murad N. Abualhasan, Mohammad Qadi, Mustafa Ghanim, Eman Massarwy, Suma Abu Ammar, Noor Zmero, Mohammad Arar, Fatima Hussein, Linda Issa, Ahmed Mousa, Abdulraziq Zarour

**Affiliations:** 1 Department of Pharmacy, Faculty of Medicine and Health Sciences, An-Najah National University, P.O. Box 7, Nablus, Palestine; 2 Department of Biomedical Sciences, Faculty of Medicine and Health Sciences, An-Najah National University, P.O. Box 7, Nablus, Palestine

**Keywords:** *Aloysia citriodora*, Essential oils, GC/MS, Antioxidant, Antibacterial, Antifungal, Cytotoxicity

## Abstract

**Background:**

*Aloysia citriodora* Palau (AC) is commonly known as Lemon Verbena and has been utilized as a medicinal tea in folkloric medicine for the treatment of abdominal spasm, anxiety, and fever. The present investigation aimed to identify the chemical ingredients of AC essential oil (EO) collected from two different locations in Palestine and to assess their antioxidant, antimicrobial, cytotoxic, and cyclooxygenase (COX) inhibitory effects.

**Methods:**

Gas chromatography/mass spectroscopy (GC/MS) technique was used to identify the chemical components of the hydro-distilled EO from both regions, while DPPH, MTS, and COX assays were utilized to estimate the antioxidant, cytotoxic, and COX inhibitory activities of the EOs, respectively. Moreover, a broth microdilution assay was used to assess antimicrobial potentials against seven microbial strains.

**Results:**

The GC/MS technique revealed the presence of 17 compounds from the AC collected from the Umm al-Fahm region and 13 compounds from the sample from the Baqa al-Gharbiyye region, while α-citral was the major component of both EOs, representing 47.62 and 43.46%, respectively. The Baqa al-Gharbiyye AC EO exerted more potent antioxidant activity than the Umm al-Fahm EO, with IC_50_ values of 11.74 ± 0.18 and 35.48 ± 0.14 μg/mL, respectively, while the positive control Trolox had antioxidant IC_50_ values of 2.45 ± 0.01 μg/mL. Interestingly, both EOs inhibited more potential activity against Methicillin-Resistant *Staphylococcus aureus* (MRSA) and *Proteus vulgaris* than Ciprofloxacin and Ampicillin antibiotics and also showed more potent antifungal activity against *Candida albicans* than Fluconazole. Moreover, the Baqa al-Gharbiyye AC EO had a more potent cytotoxic effect than the Umm al-Fahm EO, with IC_50_ values of 84.5 ± 0.24 and 33.31 ± 0.01 μg/mL, respectively, compared with Doxorubicin, which had an IC_50_ dose of 22.01 ± 1.4 μg/mL. The EOs from Baqa al-Gharbiyye showed potent activity against both COX-1 and COX-2 enzymes, with IC_50_ of 52.93 ± 0.13 and 89.31 ± 0.21 μg/mL, respectively, while the EOs from the Umm al-Fahm region showed weaker activity against these enzymes, with IC_50_ of 349.99 ± 0.33 and 1326.37 ± 1.13 μg/mL, respectively.

**Conclusion:**

Both characterized EOs have a huge variety of chemical components. The Baqa al-Gharbiyye AC EO has more potent antioxidant and cytotoxic activities than the Umm al-Fahm EO, but both have potential antimicrobial activity against MRSA, *P. vulgaris,* and *C. albicans.* These results suggest the use of AC EOs as promising sources of active ingredients in the food, cosmetic, and pharmaceutical industries.

**Supplementary Information:**

The online version contains supplementary material available at 10.1186/s12906-021-03314-1.

## Background

As other organisms strive for survival and safety from external toxic environmental conditions, plants also do their best to achieve the same purpose [1]. Plants have devised many chemical and physical defensive strategies to maintain a counterattack against herbivorous animals, viruses, parasites, and other microorganisms [2]. Chemical defensive strategies are achieved through the production of secondary metabolites, which help the plant to achieve optimum protection. Essential oils (EOs) are considered to be prominent hydrocarbon compounds and secondary metabolites produced by plants and some animals as a motif with multiple defensive employments and other functions [3].

EOs are natural compounds, extracted from various kinds of plants, and can be found in leaves, flowers, roots, woods, seeds, rhizomes, and fruits in specialized plant structures [4]. Due to the EOs’ good antiviral, anticancer, antioxidant, and antimicrobial activities, a large body of research has investigated them over the years [5]. EOs are obtained through distillation by water and steam, or by mechanical methods, such as cold pressing. Once the aromatic chemicals have been extracted, they are combined with a carrier oil to create a product that is ready for use [6, 7]. The widely diverse chemical profiles of the EOs widen the diversity of the mechanisms of action in which they are utilized and make them applicable to several kinds of industry. This is paramount, especially when considering applications concerning human health and the danger that synthetic materials can bring if used in such applications. The application of EOs can be found in the cosmetic, food, agriculture, textile, and pharmaceutical industries [8].

The production of free radicals continuously occurs in all cells and organs as part of the natural cellular function. However, excess productions of endogenous or exogenous sources of free radicals may cause many lethal diseases [9]. Natural antioxidants, obtained from vegetables and fruits, can prevent cell and tissue damage from harmful free radicals and can thus maintain optimum health in humans and other living organisms [10]. Physiologically, living cells require acceptable levels of antioxidant defense to avoid the destructive effects of the excessive production of reactive oxygen species and to avoid damage to the neurons, immune system, and many other cells [11].

The treatment of infectious diseases caused by microbial pathogens is well-documented in ancient Arabian, Chinese, and Grecian civilizations. The antibiotics discovery era started with penicillin, which was effective in controlling bacterial infections among World War II soldiers. After two decades, resistance to penicillin and other antibiotics became a substantial clinical problem. Recently, the huge and rapid distribution of microbial resistance is occurring globally, threatening the efficacy of antimicrobial agents. However, the microbial resistance problem has been ascribed to the misuse and overuse of antibiotics, in addition to the lack in discoveries of this important class of drugs [12].

Many clinical investigations have revealed that phytotherapeutics in combination with targeted-, radio-, or chemo-therapies can be utilized in cancer treatments to decrease the adverse reactions and complications and enhance the effectiveness of these treatments. Therefore, an investigation on plants is needed by scientists to explore new herbal products with anticancer properties, to help cancer patients and physicians fight this lethal disease [13].

*Aloysia citriodora* Palau (AC) is commonly known as Lemon verbena, which is a perennial herb that belongs to the Verbenaceae family and is wildly grown in the several Mediterranean, South American, and European countries [14]. This plant is well known for its characteristic aromatic odor and taste. Therefore, it is broadly utilized as an aromatic agent for the commercial production of flavoring agents and perfumes. Besides, it is widely used in the manufacture of food supplements, beverages, and various kinds of aromatic tea [15].

In fact, AC EO is prescribed for the treatment of various psychological diseases, including multiple sclerosis, nervous fatigue, depression, insomnia, stress, and anxiety. Moreover, it is also prescribed for asthma, intestinal parasites, dyspepsia, anorexia, Crohn’s disease, enterocolitis, rheumatism, tachycardia, some types of cancer and psoriasis [16, 17]. Hence, this plants species used for the treatments of many diseases related to the microbial infections, joints inflammations, oxidative stress and cancer, the current study aims to characterize the chemical composition, antioxidant, antimicrobial, anti-inflammatory and cytotoxic effects of AC EOs collected from two different locations in Palestine.

## Methods

### Plant material, chemicals, and instruments

The leaves of the wild *Aloysia citriodora* plant were collected from the Umm al-Fahm (32° 31′ 5.99“ N, 35° 09’ 7.80” E) and Baqa al-Gharbiyye (32° 25′ 7.90“ N, 35° 02’ 19.11” E) regions of historic Palestine in September 2020. The AC plant was identified by a pharmacognosist Dr. Nidal Jaradat. Collected plants were authenticated by Herbal Products Laboratory, Department of Pharmacy, An-Najah National University. A voucher specimen was deposited in the same laboratory under a deposition number (Pharm-PCT-2780). The collection of the plant material complied with the WHO Guidelines for the Assessment of Herbal Medicines and Legislation.

The collected materials were cleaned and dried in the shade at ordinary room temperature (25 ± 3 °C) and humidity (55 ± 4 RH) for 10 days. The dried materials were then coarsely grounded and kept in glass jars for further use. All chemicals were purchased from Sigma-Aldrich (Germany). A spectrophotometer-UV/Visible (Jenway® 7135, Staffordshire, UK), filter papers (Whitman No. 1, Washington, USA), shaker device (Memmert 531–25-1, Stockholm, Sweden), rotavap apparatus (Heidolph-VV 2000, Schwabach, Germany), grinder (Aero Plus 500 W Mixer Grinder, I01, Wan Chai, China), electronic-balance (Radwag, AS 220/c/2, Toruńska, Poland), freeze dryer - BT85 (Millrock Technology, China) and cryo-desiccator (Mill-rock technology, BT85, Kingston, USA) were used.

### Extraction and characterization of AC EOs

The EOs of the AC plant were extracted, utilizing the hydro-distillation procedure pronounced by Jaradat et al. [18]. Briefly, 0.1 kg of the dried leaf powder was suspended with 1 L of distilled water, and the EO was extracted using a Clevenger device operating at atmospheric pressure for 180 min at 100 °C with a hydro-distillation rate of 0.54 ml/min. The obtained AC EO was chemically dried using calcium carbonate and stored at 2 °C in the refrigerator until further use. The yields of the obtained EOs were 1.02 and 1.3% from the dried samples collected from the Umm al-Fahm and Baqa al-Gharbiyye regions, respectively.

### Qualitative and quantitative analysis

Gas chromatographic (GC) analyses were completed using an HP 5890 series II gas chromatograph equipped with a flame ionization detector (FID) and Perkin Elmer Elite-5-MS fused-silica capillary column (0.25 mm × 30 m, film thickness 0.25 μm). Helium was set at a 1.1 mL/min flow rate. The injector temperature was set at 250 °C, the oven temperature was programmed at 50 °C for 5 min followed by a ramp of 4.0 °C/min to 280 °C, and the detector (FID) was adjusted at 250 °C. The total running time was 62.50 min, and the solvent delay was from 0 to 4.0 min. The mass spectroscopy (MS) scan time was from 4 to 62.5 min, covering a mass range of 50.00 to 300.00 m/z. The mass spectra were collected under electronic ionization conditions at 70 eV [19]. In brief, retention indices (RIs) have been calculated according to the injected standard mixture of normal alkanes (C6-C27) under the mentioned conditions using the following well-known equation approved by the International Union of Pure and Applied Chemistry (IUPAC) (https://goldbook.iupac.org/terms/view/R05360). The linear temperature-programmed RIs of all the constituents were calculated from the gas chromatogram by interpolation between bracketing n-alkanes using the following equation:

$$ \mathrm{RI}=100\times \left(\left(\left(\mathrm{tR}\left(\mathrm{i}\right)-\mathrm{tR}\left(\mathrm{z}\right)\right)/\left(\mathrm{tR}\left(\mathrm{z}+1\right)-\mathrm{tR}\left(\mathrm{z}\right)\right)\right)+\mathrm{z}\right) $$

where z is the number of carbon atoms in the smaller n-alkane and tR(i), tR(z), and tR(z + 1) are the retention times of the desired compound, the smaller n-alkane, and the larger n-alkane, respectively. The identification was also confirmed by comparison of their mass spectra with those stored in the Wiley7n.l MS computer library.

### Antioxidant activity

A stock solution (1 mg/mL) was prepared from the EOs of AC leaves by dissolving 100 mg of each sample EO in 100 mL of methanol and then diluting the solution with methanol to obtain different concentrations (0, 2, 5, 7, 10, 20, 30, 40, 50, and 80 μg/mL). Then, 1 mL from each EO stock solution and 1 mL of methanol was mixed with 1 mL of DPPH solution, and the resulting solution incubated at room temperature for 30 min in a dark place together with the blank solution, which was prepared by replacing the plant EO solution with methanol. Trolox was used as a positive control, and the absorbance was measured by UV-Vis spectrophotometer at a wavelength of 517 nm, then compared with the control. The antioxidant activity of the AC EO was calculated by the following equation:

$$ \mathrm{I}\ \left(\%\right)=\left(\left[{\mathrm{ABS}}_{\mathrm{blank}}-{\mathrm{ABS}}_{\mathrm{test}}\right]/\left[{\mathrm{ABS}}_{\mathrm{blank}}\right]\right)\ast 100\% $$

where I (%) is the percentage of DPPH inhibitory activity [20, 21].

### Microbial strains, culture media, and antimicrobial activity

The antibacterial effect of AC EOs was determined using several strains of bacteria, which were obtained from the American Type Culture Collection (ATCC); *Pseudomonas aeruginosa* (ATCC 9027), *Escherichia coli* (ATCC 25922), *Klebsiella pneumonia,* (ATCC 13883), *Proteus vulgaris* (ATCC 8427), and *Staphylococcus aureus* (ATCC 25923), and from a diagnostically confirmed Methicillin-Resistant *Staphylococcus aureus* (MRSA). The antifungal activity of AC EO was evaluated against the growth of *Candida albicans* (ATCC 90028). However, the antimicrobial activity of AC EOs used in this study was estimated using the broth microdilution method.

The AC EOs were dissolved in DMSO to a concentration of 200 μg/mL. The produced solution was serially micro-diluted (2-fold) 10 times in sterile Mueller-Hinton broth. The dilution processes were performed under aseptic conditions in 96 well plates. In the micro-wells that were assigned to evaluate the antibacterial activity of AC EOs, micro-well number 11 contained plant-free Mueller-Hinton broth, which was used as a positive control for microbial growth. Micro-well number 12 contained plant-free and microbial-free Mueller-Hinton broth, which was used as a negative control for microbial growth. Micro-wells numbered 1–11 were inoculated aseptically with the test microbes. The AC EO antimicrobial activity was performed in triplicate. All the inoculated plates were incubated at 35 °C. Regarding the *Candida albicans,* the same method was used but using RPMI media instead of Mueller-Hinton broth. The incubation period lasted for about 18–24 h for those plates inoculated with the test bacterial strains and for about 48 h for those plates inoculated with *Candida albicans*. The lowest concentration of AC EO at which no visible microbial growth was observed in the micro-well was considered as the minimal inhibitory concentration (MIC) of the examined EOs. The antimicrobial activity was evaluated using known antimicrobial agents, namely Ampicillin and Ciprofloxacin, which were used as positive controls for antibacterial activity, and Fluconazole, which was used as the positive control for antifungal activity [22].

### Cell culture and cytotoxicity assay

HeLa cervical adenocarcinoma cells were cultured in RPMI-1640 media, which was supplemented with 10% fetal bovine serum, 1% Penicillin/Streptomycin antibiotics, and 1% l-glutamine. Cells were grown in a humidified atmosphere with 5% CO_2_ at 37 °C. Cells were seeded at 2.6 × 10^4^ cells/well in a 96-well plate. After 48 h, cells were incubated with various concentrations of the tested compounds for 24 h. Cell viability was assessed by CellTilter 96® Aqueous One Solution Cell Proliferation (MTS) Assay according to the manufacturer’s directions. Briefly, at the end of the treatment, 20 μL of MTS solution per 100 μL of media was added to each well and incubated at 37 °C for 2 h. Absorbance was measured at 490 nm [23].

### Biological cyclooxygenase (COX) assay method

The ability of the EOs to prevent the conversion of arachidonic acid to PGH2 by bovine COX-1 and human recombinant COX-2 was assessed using a cyclooxygenase (COX) inhibitor screening assay kit (Item No: 560131) according to the Cayman chemical manufacturer’s guidelines (USA). The 50% inhibitory concentration (IC_50_) of COX-1/COX-2 activity of the compounds was carried out, with the assay run, in duplicate, with two concentrations (350 and 50 μg/mL). A standard curve of eight concentrations of prostaglandin, a non-specific binding sample, and a maximum binding sample was used, as instructed in the kit manual, to determine the inhibition of the EO samples, applying the generated multiple regression best-fit line. The percentage inhibition of the two concentrations was used to calculate the IC_50_ [24].

### Statistical analysis

The conducted tests were performed in triplicate for the AC EOs. The results were expressed as means (±) standard deviation (SD). Statistical Package for the Social Sciences (SPSS) statistical package was used to calculate statistical difference. A *p*-value < 0.05 was considered statistically significant.

## Results

**Fig. 1:**
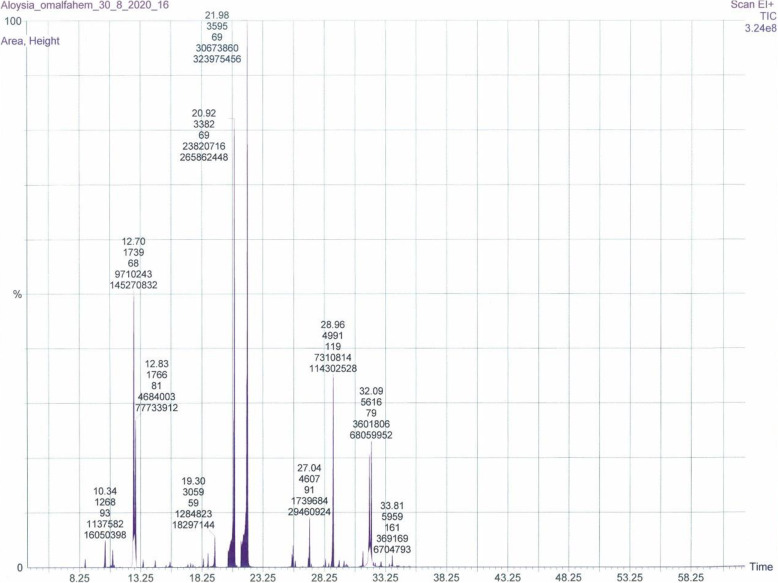
GC-MS chromatogram of (AC) EO from Umm al-Fahm

**Fig. 2:**
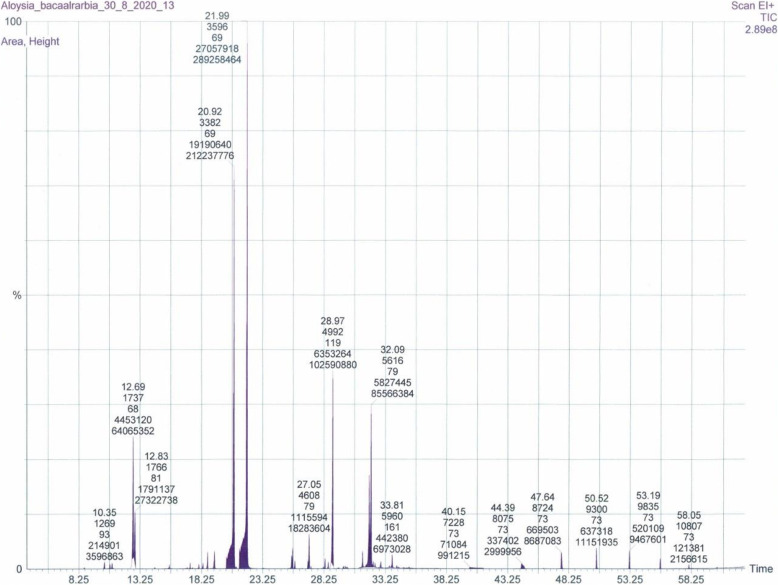
GC-MS chromatogram of (AC) EO from Baqa al-Gharbiyye

The GC-MS characterization of the EO extracted from the leaves of AC plants collected from two regions of Palestine revealed the presence of 17 compounds from the sample collected from the Umm al-Fahm region and 13 compounds from the AC plant collected from the Baqa al-Gharbiyye region, both representing 100% of the total mass, it was showed in Table 1 (Additional file 1), and Figs. 1 and 2.

### Antioxidant activity

**Fig. 3:**
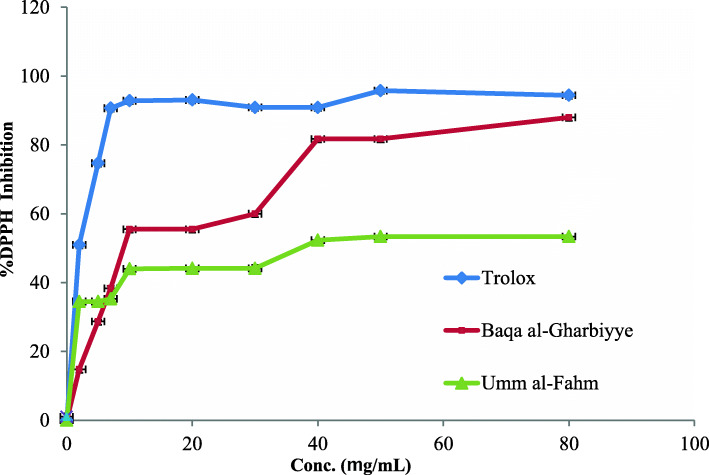
The DPPH inhibitory potentials by the (AC) EOs from Umm al-Fahm and Baqa al-Gharbiyye regions and the positive control Trolox

The ability of the EOs isolated from the AC leaves from the Umm al-Fahm and Baqa al-Gharbiyye regions to neutralize free radicals was assessed utilizing a DPPH assay. Figure 3 depicts the IC_50_ values and DPPH inhibitory potentials for the AC EOs and the results showed that both EOs have potential free radical scavenging properties compared with the standard antioxidant preparation Trolox.

### Antimicrobial activity

The broth microdilution assay was used to evaluate the antimicrobial effects of the AC EOs from the Umm al-Fahm and Baqa al-Gharbiyye regions and the positive controls Ampicillin, Ciprofloxacin, and Fluconazole. Table 1 showed that both EOs have the same antibacterial effect against MRSA, *S. aureus*, *K. pneumoniae*, and *P. vulgaris*, while these EOs were inactive against *E. coli* and *P. aeruginosa*. Moreover, both oils have antifungal activity against *C. albicans.*

**Table 1:** MIC values (μg/ml) of (AC) essential oils, Ampicillin, Ciprofloxacin and Fluconazole

Tested samples	Microbial strains						
	MRSA	***S. aureus***	***E. coli***	***K. pneumoniae***	***P. vulgaris***	***P. aeruginosa***	***C. albicans***
EO from Baqa al-Gharbiyye	2.5	2.5	R	5	2.5	R	0.625
EO from Umm al-Fahm	2.5	2.5	R	5	2.5	R	0.312
Fluconazole	–	–	–	–	–	–	1.56
Ampicillin	R	3.12	3.12	1	18	R	–
Ciprofloxacin	12.5	0.78	1.56	0.125	15	3.12	–

*R* Resistance

### Cytotoxicity

**Fig. 4:**
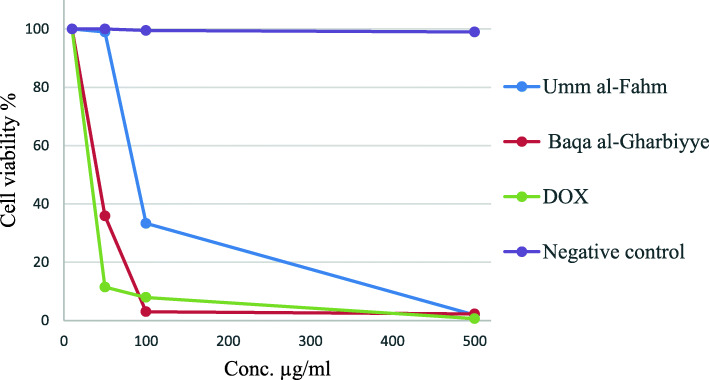
Cytotoxic effects of the EOs obtained from the (AC) plant collected from Umm al-Fahm and Baqa al-Gharbiyye regions, positive control (Dox) and negative controls

According to the MTS assay results, both screened EOs have cytotoxic effects against cervical cancer cells (HeLa) in a dose-dependent manner, the HeLa cancer cell viability of AC EOs was presented in Fig. 4 in comparison with positive control doxorubicin (DOX). The AC EOs from the Umm al-Fahm and Baqa al-Gharbiyye regions showed a cytotoxic effect, and according to the inhibition activity results the IC_50_ values were 84.5 ± 0.24 and 33.31 ± 0.01 μg/mL, respectively.

### Cyclooxygenase (COX) inhibition activity

The AC EOs from Umm al-Fahm and Baqa al-Gharbiyye were evaluated on COX enzymes, and their activities were compared with the positive control drug ketoprofen. Both EOs showed inhibitory activity toward COX-1 and COX-2 enzymes, and they showed a better selectivity ratio than ketoprofen (> 0.20). However, the EOs from Baqa al-Gharbiyye showed better activity against both enzymes compared with the EOs from Umm al-Fahm, and the activity on the COX-1 enzyme was more potent than the activity on the COX-2 enzyme, as presented in Table 2.

**Table 2:** IC_50_ inhibition of COX-1 and COX-2 and COX-2 inhibition selectivity of the EOs

Name	IC_50_ (μg/ml)		Selectivity ratio for COX-2
	COX-1	COX-2	
Ketoprofen	7.8827 ± 0.02	40.1762 ± 0.12	0.20
Umm al-Fahm	349.988 ± 0.33	1326.378 ± 1.13	0.26
Baqa al-Gharbiyye	52.9349 ± 0.13	89.3088 ± 031	0.59

## Discussion

EO plant products have been used as a human pharmacy for thousands of years and are considered an endless source of medicines [25, 26]. The interest in aromatherapy and medicinal natural EOs starts to increase widely as an alternative therapy for a range of various health disorders.

### Phytochemistry

Slight differences in yields and phytochemical compositions of EOs of the AC plant from Umm al-Fahm and Baqa al-Gharbiyye regions was observed. However, 1.02% yield was recorded for the EO obtained from Umm al-Fahm plant while 1.3% was obtained from the Baqa al-Gharbiyye sample. The GC-MS phytochemical characterization of the EOs obtained by the hydrodistillation method from the leaves of AC plants from Umm al-Fahm and Baqa al-Gharbiyye, revealed that geranial (α-citral) was the major component of both EOs, representing 47.62 and 43.46%, respectively while the second major component was trans-1,2-Bis-(1-methylethenyl) cyclobutene (15.07%) and α-curcumene (14.39%), respectively.

The most represented chemical classes were oxygenated monoterpenoid (56.89%), hydrocarbon monoterpene (18.58%), and hydrocarbon sesquiterpene (18.1%) in the AC plant EO from Umm al-Fahm region. While, the major classes from Baqa al-Gharbiyye sample were oxygenated monoterpenoid (48.89%), hydrocarbon sesquiterpene (31.7%), and hydrocarbon monoterpene (10.16%).

A study conducted by Gil et al. found that the genotype EO of AC collected from the Rancagua region of Argentina was geranial (α-citral) and represented 21.3% of the total EOs [14]. Another investigation of the AC EO from the Agadir region of Morocco by Oukerrou et al. found that the major EO ingredients were trans-caryophyllene oxide, 훽-spathulenol, and curcumene, representing 13.52, 13.27, and 11.47% of the total EO, respectively [27]. An investigation conducted by Farahmandfar et al. found that the major components of AC EO are limonene (18.41 ± 1.24%), neral (16.1 ± 2.97%), and geranial (13.02 ± 1.34%) [28].

Moreover, Argyropoulou et al. from Greece investigated the chemical components of the EO extracted from the leaves of AC and identified 43 molecules, representing 97.8% of the total EO. However, geranial, neral, and limonene (38.7, 24.5, and 5.8%) were found to be the major ingredients [29].

### Antioxidant activity

The DPPH radical scavenging assay is a popular in-vitro technique used to estimate the ability of EOs and plant extracts to neutralize the free radicals caused by the DPPH reagents. In the current study, this colorimetric assay was used to assess the antioxidant potential of the EOs extracted from AC leaves collected from the Baqa al-Gharbiyye and Umm al-Fahm regions. The results showed that these EOs have potential DPPH inhibitory activity comparing with Trolox. Essentially, Baqa al-Gharbiyye EO has the strongest antioxidant activity compared with the EO from the Umm al-Fahm region, with antioxidant IC_50_ values of 11.74 ± 0.18 and 35.48 ± 0.14 μg/mL, respectively, compared with Trolox, which had an antioxidant IC_50_ value of 2.45 ± 0.01 μg/mL.

An investigation conducted by Hosseini et al. found that AC EO has potent antioxidant activity compared with the positive control (BHT), with IC_50_ values of 11.33 ± 01 and 27.43 ± 04 μg/mL, respectively [30]. The presence of heteroatom-containing compounds in the EOs can induce antioxidant activity, Oxygen containing moieties like phenols or hydroxyl have antioxidant activity more potent than Nitrogen-containing structures like aniline [31], However, Citral is the main component of various plants was reported as antioxidant agents [32] and in our EOs of AC one of the main component was the α-Citral from both regions, as well as more chemical components with Oxygen atom-like Methyl lineoleate and [2,2-Dimethyl-4-(3-methyl but-2-enyl)-6-methylidenecyclohexyl] methanol was observed in the EOs of AC from Baqa al-Gharbiyye in quantities two-three times more than Umm al-Fahm EOs, and this can explain the antioxidant activity of both EOs and the more potent activities regarding Baqa al-Gharbiyye than Umm al-Fahm EOs.

### Antimicrobial effect

New antimicrobial drugs are urgently required to solve the problem of increasing rates of global microbic resistance. The antimicrobial results of the current study revealed that AC EOs from Baqa al-Gharbiyye and Umm al-Fahm have strong anti-MRSA activity, with a MIC value of 2.5 μg/mL compared with Ampicillin, which has resistance against MRSA, and with Ciprofloxacin, which has a MIC value of 12.5 μg/mL. In addition, the EOs showed strong potential activity against the *P. vulgaris* strain, with a MIC value of 2.5 μg/mL compared with Ampicillin, which has resistance against *P. vulgaris,* and with Ciprofloxacin, which has a MIC value of 3.12 μg/mL. They also showed more antibacterial potential activity against *S. aureus* than Ampicillin, with MICs of 2.5 and 3.12 μg/mL, respectively. Finally, the AC EOs from Baqa al-Gharbiyye and Umm al-Fahm had more potential anticandidal activity than Fluconazole, with MIC doses of 0.625, 0.312, and 1.56 μg/mL, respectively.

Oukerrou et al. found that AC EO inhibited the growth of *E. coli* and *S. aureus,* with MIC values of 8.37 and 5.84 mg/mL, respectively [27]. Another study conducted by Hosseini et al. evaluated the antibacterial activity of AC EO using the microdilution method and found that it has antibacterial activity against *E. coli*, *P. aeruginosa,* and *S. aureus,* with MICs of 2500, 2500, and 1250 μg/mL, respectively [30]. Among the EO compounds, the aromatic containing compounds like carvacrol, thymol, eugenol, and cinnamaldehyde appear to have interesting antimicrobial activities as well as the aliphatic components, like nerol, linalool, citral, geraniol, perillaldehyde, and α-terpineol, possess potent antimicrobial activities [33], the antibacterial mechanism of these aromatic and aliphatic compounds is that in their lipophilic ability to partition in the lipophilic lipids of the mitochondria and cytoplasmic membrane as well as they could disturb the structures, and resulting in leakage of bacterial cell contents [34]. In this work, the presence of a high percentage of lipophilic structures like α-citral and α-Curcumene could be the main reason for antibacterial activities.

### Cytotoxic activity

The MTS assay revealed that the AC EOs from the Umm al-Fahm and Baqa al-Gharbiyye regions exerted a dose-dependent cytotoxic effect on HeLa tumor cell lines. However, the Baqa al-Gharbiyye AC EO had a more potent cytotoxic effect than the Umm al-Fahm EO, with IC_50_ values of 84.5 ± 0.24 and 33.31 ± 0.01 μg/mL, respectively, and compared with Doxorubicin, which has a cytotoxic effect against HeLa cancer cells with an IC_50_ dose of 22.01 ± 1.4 μg/mL. In fact, the viability was over 98.13 and 96.09% at the concentration of 500 μg/mL, respectively, to induce tumor cell lysis.

An investigation conducted by Oukerrou et al. found that AC EO exerted a dose-dependent cytotoxic effect on P815, MCF7, and VERO tumor cell lines, with IC_50_ ranging from 6.60 to 79.63 μg/mL [27]. Moreover, Chaves-Quirós found that Citral, which is a major component in the current study, has a cytotoxic effect against human periodontal ligament fibroblast cancer cells and exhibited bacteriostatic/bactericidal effects to *Streptococcus mutans, Lactobacillus rhamnosus,* and *Enterococcus faecalis* strains [35].

To put it more simply, citral is a flavoring agent that is widely used in the fragrance, beverage, and food industries which is an aldehydic monoterpenoid usually present in the form of the stereoisomer neral [36]. Citral has been shown to damage the cell membrane of microbial pathogens by decreasing the intracellular ATP concentration, reducing pH_in_, and causing cell membrane hyperpolarization [37]. Several studies have demonstrated that citral has potential anti-inflammatory [38], anti-corrosive [39], and antimicrobial effects [40]*.*

Further in vivo pharmacological, taxological, and pharmacokinetic evaluations must be conducted on AC EOs to understand the mechanism of action of this oil and also to estimate its anticancer effect on animal models to establish its possible use in clinical trials on human subjects.

### Cyclooxygenase (COX) inhibition activity

The COX enzymes were identified as therapeutic targets of non-steroidal anti-inflammatory drugs (NSAIDs). The discovered agents working by blocking the biosynthesis of prostaglandins (PGs), which have different physiological and pharmacological functions [41]. The EO from Baqa al-Gharbiyye showed better activity against both COX-1 and COX-2 enzymes, with IC_50_ of 52.93 ± 0.13 and 89.31 ± 0.21 μg/mL, respectively, while the EO from the Umm al-Fahm region showed weaker activity against these enzymes, with IC_50_ of 349.99 and 1326.37 μg/mL, respectively. However, both EOs showed a better selectivity ratio toward the COX-2 enzyme than ketoprofen, with a ratio of more than 0.20, especially the EO from Baqa al-Gharbiyye where the ratio was 0.59. The significant differences in the activities of each EO could be due to the presence of methyl lineoleate, α-curcumene, and 1-methylene-2b-hydroxymethyl-3,3-dimethyl-4b-(3-methylbut-2-enyl)-cyclohexane in the Baqa al-Gharbiyye EO in a greater percentage than in the EO from Umm al-Fahm; the chemical components such as methyl lineoleate have a chemical structure similar to arachidonic acid, which is the main substrate of the COX enzymes, and α-curcumene has a phenyl ring which is considered as one of the main pharmacophore chemical structures of NSAIDs.

## Conclusion

The results obtained in this investigation revealed that the chemical profile of AC EOs grown in Palestine is slightly variable depending on the region where the plant was collected. Antioxidant estimations showed that the AC EO from Baqa al-Gharbiyye has potential antioxidant activity almost three-fold higher than the EO from Umm al-Fahm. Both EOs were even more microbiostatic against *P. vulgaris* and *C. albicans* than the positive controls used, Ampicillin, Ciprofloxacin, and Fluconazole. On the other hand, both screened AC EOs possesses potential cytotoxic effects against HeLa tumor cell lines. In fact, the Baqa al-Gharbiyye AC EO has a more potent cytotoxic effect than the Umm al-Fahm EO by two and a half-fold. Obviously, the EO of AC leaves collected from Baqa al-Gharbiyye has potent cytotoxic activity compared with Doxorubicin. The EO from Baqa al-Gharbiyye showed better activity against both COX-1 and COX-2 enzymes, while both EOs have a better selectivity ratio towards COX-2 enzyme than the positive control ketoprofen drug. These findings indicate that the AC EO collected from both regions are promising natural sources of antioxidant, antimicrobial, and antitumor formulations.

## Supplementary Information

**Additional file 1: Table S1.** The chemical components of the (AC) plant EOs collected from Umm al-Fahm and Baqa al-Gharbiyye regions.

## Data Availability

The datasets used and/or analysed during the current study are available from the corresponding author on reasonable request. The datasets supporting the conclusions of this article are included in the manuscript. The raw data and materials of the current study are available from the corresponding author on reasonable request.

## Abbreviations

AC: *Aloysia citriodora*
COX: Cyclooxygenase
NSAIDs: Non-steroidal anti-inflammatory drugs
IC_50_: 50% Inhibition concentration
DMSO: Dimethyl sulfoxide
DPPH: 2,2-diphenyl-1-picrylhydrazyl
Trolox: 6-hydroxy-2,5,7,8-tetramethylchroman-2-carboxylic acid
A_B_: Absorbance of the blank solution
A_ts_: Absorbance of the tested sample solution
HeLa: Human cervix adenocarcinoma cell line
Dox: Doxorubicin
Conc.: Concentration
