# Supplementary material for: Spectral characterization, antioxidant, antimicrobial, cytotoxic, and cyclooxygenase inhibitory activities of Aloysia citriodora essential oils collected from two Palestinian regions
Source: BMC Complement Med Ther. 2021 May 17;21:143. doi: 10.1186/s12906-021-03314-1 (PMC8130314; doi:10.1186/s12906-021-03314-1)
Supplement: Supplementary file 1 — Additional file 1: Table S1. The chemical components of the (AC) plant EOs collected from Umm al-Fahm and Baqa al-Gharbiyye regions. [file 12906_2021_3314_MOESM1_ESM.docx]

**Table 1** The chemical components of the (AC) plant EOs collected from Umm al-Fahm and Baqa al-Gharbiyye regions

| **Common name** | **Formula** | **Chemical structure** | **Retention time** | **Retention index** | **EOs %, Umm al-Fahm,** | **EOs %, Baqa al-Gharbiyye** |
| --- | --- | --- | --- | --- | --- | --- |
| α-Phellandrene | C_10_H_16_ |  | 8.7 | R:785 | - | 0.08 |
| β-Ocimene | C_10_H_16_ | **** | 8.72 | R:847 | 0.42 | - |
| β-Phellandrene | C_10_H_16_ |  | 10.34 | R:870 | 1.77 | - |
| Vinyl amyl carbinol | C_8_H_16_O |  | 10.79 | R:759 | - | 0.32 |
| Octanal, (2,4-dinitrophenyl) hydrazone | C_14_H_26_N_4_O_4_ |  | 10.95 | R:712 | 1.00 | - |
| trans-1,2-Bis-(1-methylethenyl)cyclobutane | C_10_H_16_ |  | 12.7 | R:895 | 15.07 | 10.08 |
| Eucalyptol | C_10_H_18_O |  | 12.83 | R:832 | 7.28 | 4.06 |
| Β-terpinene | C_10_H_16_ |  | 14.43 | R:839 | 0.39 | - |
| 3,7,11-Trimethyl-3-hydroxy-6,10-dodecadien-1-yl acetate | C_17_H_30_O_3_ |  | 15.64 | R:763 | 0.35 | 0.3 |
| Phenylacetic acid, 2-methylcyclohex-2-enyl ester | C_15_H_18_O_2_ |  | 18.36 | R:837 | 0.42 | - |
| α-Terpineol | C_10_H_18_O |  | 19.3 | R:901 | 1.99 | 1.37 |
| α-Citral | C_10_H_16_O |  | 21.4 | R:843 | 47.62 | 43.46 |
| 8-Methylidenebicyclo[5.1.0]octane | C_9_H_14_ |  | 25.69 | R:778 | 0.93 | - |
| Caryophyllene | C_15_H_24_ |  | 27.04 | R:886 | 2.7 | 2.53 |
| Aromadendrene | C_15_H_24_ |  | 28.34 | R:810 | - | 0.58 |
| α-Curcumene | C_15_H_22_ |  | 28.97 | R:893 | 11.35 | 14.39 |
| [2,2-Dimethyl-4-(3-methylbut-2-enyl)-6-methylidenecyclohexyl]methanol | C_15_H_26_O |  | 29.7 | R:910 | 2.7 | 13.2 |
| α-Farnesene | C_15_H_24_ |  | 31.39 | R:745 | 0.78 | - |
| Methyl lineoleate | C_19_H_34_O_2_ |  | 31.93 | R:825 | 4.66 | 8.63 |
| γ-Muurolene | C_15_H_24_ |  | 33.81 | R:857 | 0.57 | 1.00 |
| Total | | | | | 100.00 | 100.00 |
| **Phytochemical classes** | | | | | **Umm al-Fahm, %** | **Baqa al-Gharbiyye, %** |
| Hydrocarbon monoterpene | | | | | 18.58 | 10.16 |
| Oxygenated monoterpenoid | | | | | 56.89 | 48.89 |
| Hydrocarbon sesquiterpene | | | | | 18.1 | 31.7 |
| Oxygenated sesquiterpenoid | | | | | 0.77 | 0.3 |
| Others | | | | | 5.66 | 8.95 |
| **Total** | | | | | 100.00 | 100.00 |
